# Multidrug-resistant *Corynebacterium diphtheriae* in people with travel history from West Africa to France, March to September 2023

**DOI:** 10.2807/1560-7917.ES.2023.28.46.2300615

**Published:** 2023-11-16

**Authors:** Sylvie Brémont, Virginie Passet, Mélanie Hennart, Laure Fonteneau, Julie Toubiana, Edgar Badell, Sylvain Brisse

**Affiliations:** 1 Institut Pasteur, Université Paris Cité, Biodiversity and Epidemiology of Bacterial Pathogens, Paris, France; 2 Institut Pasteur, National Reference Center for Corynebacteria of the Diphtheriae Complex, Paris, France; 3 Santé publique France, Saint-Maurice, France; 4 General pediatric and infectious diseases, Necker-enfants malades hospital, Université Paris Cité, Paris, France

**Keywords:** Diphtheria, outbreaks, antimicrobial resistance, genomic epidemiology, Africa

## Abstract

We describe 10 unlinked cases of *Corynebacterium diphtheriae* infection (nine cutaneous, one respiratory) in France in 2023 in persons travelling from Guinea, Mali, Senegal, Niger or Nigeria and Central African Republic. Four isolates were toxigenic. Seven genomically unrelated isolates were multidrug-resistant, including a toxigenic respiratory isolate with high-level resistance to macrolides and beta-lactams. The high rates of resistance, including against first-line agents, call for further microbiological investigations to guide clinical management and public health response in ongoing West African outbreaks.

While diphtheria is largely controlled in high-income countries, regions with suboptimal vaccination coverage can experience large outbreaks, and travel from endemic or epidemic regions may result in case importation [1-4]. Whereas classical diphtheria presents as respiratory infection caused by *Corynebacterium diphtheriae*, cutaneous infections are common and play an important role in transmission [5]. Here we report on 10 unlinked cases of *Corynebacterium diphtheriae* infection (nine cutaneous, one respiratory) in France in 2023 in persons travelling from Guinea, Mali, Senegal, Niger or Nigeria and Central African Republic.

## Diphtheria surveillance at the national reference laboratory

In France, diphtheria is a mandatory notifiable disease and isolates are submitted to the national reference laboratory (NRL) at Institut Pasteur, Paris together with information on sample type, demographics and travel history of the patients and clinical presentation. Diagnosis at the NRL relies on a multiplex quantitative PCR (qPCR) assay for detection of the *tox* gene coding for diphtheria toxin and for identification of *C. diphtheriae* and *C. ulcerans* [6]. Production of the toxin is determined from isolated colonies using Elek’s immunoprecipitation test [7].

## Isolates submitted to the national reference laboratory between March and September 2023

Between 30 March and 30 September 2023, the NRL received and analysed 12 *C. diphtheriae* isolates from 12 cases with a travel history in West Africa (11 isolates) and Central African Republic (one isolate) (Table 1). Time between entry into France and sampling ranged from 5 to 31 days. All isolates had been identified as *C. diphtheriae* by MALDI-TOF mass spectrometry (Bruker, Germany) before being sent to the NRL, where identification was confirmed by qPCR. Three isolates were linked in a single household and had the same genome sequence, only the index case was included in further analysis. The 10 deduplicated isolates were characterised using biovar determination, antimicrobial susceptibility testing (AST) and genomic sequencing (Nextera genomic library and Illumina NextSeq-500 chemistry) as described [8,9]. Methods for AST and interpretation of the results followed the European Committee on Antimicrobial Susceptibility Testing (EUCAST) 2023 recommendations [10] and for some of them Antibiogram Committee of the French Society of Microbiology recommendations [11,12] (Table 2).

**Table 1:** Characteristics of *Corynebacterium diphtheriae* isolates from people with travel history to West Africa, France, March–September 2023 (n = 10)^a^

Characteristics	Isolate identification number																				
	FRC1894		FRC1913		FRC1956		FRC1958		FRC1973		FRC1980		FRC1984			FRC1987		FRC1964		FRC2000	
Clinical form	Cutaneous		Cutaneous		Cutaneous		Cutaneous		Cutaneous		Cutaneous		Respiratory			Cutaneous		Cutaneous		Cutaneous	
Country (recent travel)^b^	Niger and Nigeria		Guinea		Mali		Mali		Guinea		Senegal		Guinea			Senegal		Central African Republic		Mali	
ST (MLST)	ST830		ST913		ST103		ST935		ST377		ST940		ST941			ST942		ST377		ST183	
ST (core genome MLST)	2981		3014		3008		3000		3001		2685		3012			3016		3004		3025	
Genomic cluster	918		939		935		930		931		868		938			940		71		943	
*tox* gene	Positive		Negative		Negative		Negative		Positive		Negative		Positive			Negative		Negative		Positive	
Toxin production (Elek’s test)	Positive		NA		NA		NA		Positive		NA		Positive			NA		NA		Positive	
*spuA* gene^c^	Negative		Negative		Negative		Negative		Negative		Negative		Positive			Negative		Negative		Negative	
**Antimicrobial agent**	**Resistance phenotype and MIC (mg/L) if the isolate was resistant**																				
	SIR	MIC	SIR	MIC	SIR	MIC	SIR	MIC	SIR	MIC	SIR	MIC	SIR	MIC		SIR	MIC	SIR	MIC	SIR	MIC
Ciprofloxacin	I		R	6	I		R	6	R	> 32	I		I			I		R	> 32	I	
Moxifloxacin	S		R	1.5	S		R	1.5	R	6	S		S			S		R	6	S	
Erythromycin	S		S		R	0.38	S		S		S		R	16		S		S		S	
Clindamycin	S		S		S		S		S		S		R	> 256		S		S		S	
Azithromycin	S		R	32	R	12	S		S		S		R	> 256		S		S		S	
Clarithromycin	S		S		S		S		S		S		R	8		S		S		S	
Pristinamycin	S		S		S		S		S		S		S			S		S		S	
Spiramycin	S		R		R		S		S		I		R			S		S		S	
Tetracycline	R	16	R	4	R	24	R	32	R	6	S		R	32		S		R	6	S	
Linezolid	S		S		S		S		S		S		S			S		S		S	
Rifampicin	S		R	> 32	S		S		R	> 32	S		R	> 32		S		S		S	
Vancomycin	S		S		S		S		S		S		S			S		S		S	
Gentamicin	R	1.5	R	1	R	1	R	1.5	R	1.5	R	1.5	S			S		R	2	R	2
Cefotaxime	I		I		I		I		I		I		R	4		I		I		I	
Penicillin	I		R	1.5	R	2	R	1.5	I		I		R	> 256		R	1.5	I		I	
Oxacillin	S		S		R	48	S		S		S		R	> 256		R	24	S		S	
Amoxicillin	S		S		S		S		S		S		R	64		S		S		S	
Trimethoprim-sulfamethoxazole	R	> 32	R	> 32	R	3	R	4	R	2	R	0.75	S			R	> 32	R	1.5	R	8
Sulfonamide^d^	R		R		S		R		R		R		S			R		R		R	
Trimethoprim	R	> 32	R	> 32	R	> 32	R	> 32	R	> 32	S		S			R	> 32	R	> 32	I	
Meropenem	S		S		S		S		S		S		R		4	S		S		S	
Fosfomycin, used as control	R		R		R		R		R		R		R			R		R		R	
Number of resistances to distinct classes	3		7		5		5		5		2		5			2		4		2	
Resistance genes and context^e^	sul1, qacEdelta1, dfrA15 || cmx || tet(O)		dfrA16, qacL, qacEdelta1, sul1, tet(33) || erm(X), pbp2m || cmx		qacE, sul1 || pbp2m, erm(X) || dfrA1, aadA15 || tet(O) || cmx		pbp2m || sul1 || tet(O)		aph(3'')-Ib;aph(6)-Id || gyrA_D93A, gyrA_S89F || sul1 || cmx || tet(33) || aph(3')-Ia		sul1		pbp2m, erm(X), cmx || tet(O)			sul1, qacEdelta1, qacL, dfrA16 || pbp2m		gyrA_D93A, gyrA_S89F || aph(6)-Id;aph(3'')-Ib || sul1 || cmx || tet(33) || aph(3')-Ia		sul1	

MIC: minimum inhibitory concentration; MLST: multilocus sequence typing; NA: not applicable; SIR: susceptible, intermediate, resistant; ST: sequence type.

^a^ Three isolates were linked in a single household and had the same genome sequence, only the index isolate was included in further analysis. Therefore, 10 isolates were included in the analysis and not 12.

^b^ Time between entry into France and sampling ranged from 5 to 31 days.

^c^ *Corynebacterium diphtheriae* biovar Gravis marker.

^d^ No Etest was available for sulfonamide.

^e^ Double pipes (||) separate genes or markers located in distinct genomic regions.

**Table 2:** Antimicrobial agents used and breakpoints applied for testing *Corynebacterium diphtheriae* isolates from people with travel history to West Africa, France, 2023 (n = 10)

Antimicrobial agent	Class	S	I	R	Interpretation criteria	Comment
Gentamicin	Aminoglycoside	≥ 23		< 23	[12]	
Spiramycin	Macrolide	≥ 24	19–23.99	< 19	[11]	
Amoxicillin	Beta-lactam	≥ 23	16–22.99	< 16	[11]	
Cefotaxime	Beta-lactam	≥ 50	15–49.99	< 15	[10]	5 mg disks were used
Oxacillin	Beta-lactam	≥ 20		<20	[11]	
Penicillin	Beta-lactam	≥ 50	12–49.99	<12	[10]	1 IU disks were used
Meropenem	Carbapenem	≥ 24		<24	[10]	
Tetracycline	Cycline	≥ 24		<24	[10]	Interpretation is applicable to doxycycline
Sulfonamide	Folate pathway inhibitors	≥ 17	12–16.99	< 12	[11]	
Trimethoprim	Folate pathway inhibitors	≥ 16	12–15.99	< 12	[11]	
Trimethoprim-sulfamethoxazole	Folate pathway inhibitors	≥ 23		< 23	[10]	
Fosfomycin	Fosfomycin	≥ 14		< 14	[11]	Control, resistance expected
Vancomycin	Glycopeptide	≥ 17		< 17	[11]	
Azithromycin	Macrolide	≥ 22	17–21.99	< 17	[11]	
Clarithromycin	Macrolide	≥ 22	19–21.99	< 19	[11]	
Clindamycin	Macrolide	≥ 15		< 15	[10]	Only applicable to *C. diphtheriae*, not *C. ulcerans*
Erythromycin	Macrolide	≥ 24		< 24	[10]	
Linezolid	Oxazolidinones	≥ 25		< 25	[10]	
Pristinamycin	Macrolide	≥ 22	19–21.99	< 19	[11]	
Ciprofloxacin	Quinolone	≥ 50	24–49.99	< 24	[10]	
Moxifloxacin	Quinolone	≥ 24	21–23.99	< 21	[11]	
Rifampicin	Rifamycin	≥ 24		< 24	[11]	

I: susceptible at higher exposure; IU: international unit; R: resistant; S: susceptible.

## Demographic and clinical characteristics of cases

All isolates except one were from cutaneous infections. Four cases had cutaneous infections in leg wounds and one had a Buruli ulcer. Four isolates were *tox*-positive by qPCR and produced the toxin phenotypically, as demonstrated by Elek’s test. Two cases, infected with *tox-*positive strains, were hospitalised. No deaths were reported. Cases seeked healthcare in six French administrative districts (’Régions‘) and were aged 1–67 years (median 21). Sex-ratio was 1:1. A recent travel history from Guinea (n = 3), Mali (n = 3), Senegal (n = 2), Central African Republic (n = 1) and Niger and Nigeria (n = 1) was documented. For six cases, vaccination status was unknown, three were not vaccinated and only one (aged 1 year) was vaccinated.

## Multilocus sequence types

The 10 deduplicated isolates corresponded to nine multilocus sequence typing (MLST) sequence types, as ST377 was found in two cases, linked to Central African Republic and Guinea (Table 1). However, these two isolates belonged to two distinct genomic clusters by core genome MLST (Table 1). The high level of genomic diversity showed that the 10 isolates were not closely related phylogenetically and highlighted a multiclonal bacterial population. All isolates were biochemically characterised as biovar Mitis except the respiratory isolate, which was biovar Gravis, consistent with the presence in its genome of the *spuA* gene (Table 1).

## Antimicrobial susceptibility testing

**Figure 1:**
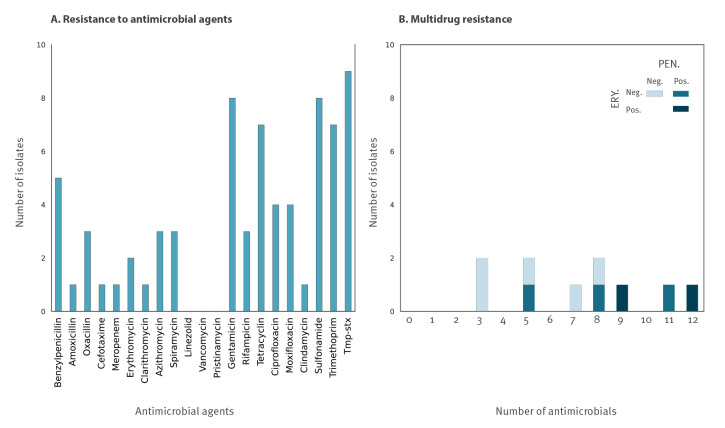
Phenotypic resistance and multidrug resistance of *Corynebacterium diphtheriae* isolates from patients with travel history to West Africa, France, 2023 (n = 10)

ERY: erythromycin; PEN: penicillin; Tmp-stx: trimethoprim-sulfamethoxazole.

Antimicrobial susceptibility testing showed resistance against most antimicrobial agents (Figure 1A). Half or more of the isolates were resistant to folate pathway inhibitors, gentamicin, tetracycline and, more worryingly, benzylpenicillin. However, all except one were susceptible to amoxicillin, the recommended antimicrobial in France. Defining multidrug resistance as resistance to agents in three or more classes (discarding fosfomycin, to which *Corynebacterium* species are intrinsically resistant), seven of the 10 isolates were multidrug-resistant. The 10 isolates were resistant to 3–12 individual agents (Figure 1B), considering the 21 antimicrobial agents tested (excluding fosfomycin). Five isolates were resistant to benzylpenicillin and two of these were additionally resistant to erythromycin (three to azithromycin), i.e. to both first-line recommended treatments.

**Figure 2:**
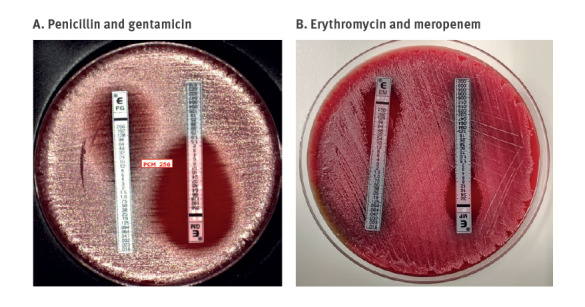
Heteroresistance of *Corynebacterium diphtheriae* isolate FRC1984 against benzylpenicillin and erythromycin, France, 2023

Whereas three *tox*-positive isolates only had resistance to antimicrobial agents of secondary clinical relevance (tetracycline, folate pathway inhibitors, aminoglycosides and/or quinolones) (Table 1), one *tox*-positive isolate (FRC1984, linked to Guinea) had high-level resistance to benzylpenicillin and oxacillin (minimum inhibitory concentration (MIC) > 256 mg/L), amoxicillin (MIC 64 mg/L), erythromycin (MIC 16 mg/L) and azithromycin (MIC > 256 mg/L). Tetracycline and rifampicin were also inactive against this isolate, which, however, remained susceptible at increased exposure to ciprofloxacin. Surprisingly, the isolate was also resistant to meropenem (MIC 4 mg/L). However, we noted that this isolate presented a heterogeneous resistance to penicillin, with a characteristic halo denoting higher susceptibility in parts of the bacterial population (Figure 2A). High-level resistance to beta-lactams and carbapenems, associated with heteroresistance, was previously reported only once, to our knowledge [13]. Here, additionally, we also observed heteroresistance to erythromycin (Figure 2B).

E: erythromycin; GM: gentamicin; MP: meropenem; PG: benzylpenicillin.

A: Etest gradient strip with benzylpenicillin (PG) and gentamicin (GM). Note the heterogeneous resistance to penicillin.

B: Etest gradient strip with erythromycin (EM) and meropenem (MP). Note the small colonies growing in vicinity of the erythromycin gradient strip at higher concentrations than the main population.

Genomic sequencing disclosed antimicrobial resistance genes or mutations that were highly consistent with phenotypes (Table 1). Gene *pbp2m* (also named *pbp2C* by Forde et al. 2021 [13]) was found in five isolates, all showing low-level resistance to benzylpenicillin (MIC 1.5 or 2 mg/L) except isolate FRC1984 (see above). The macrolide-resistance gene *ermX* was found in the three macrolide-resistant isolates (travel from Guinea, Mali and Niger), always co-located with *pbp2m*. This physical association was previously reported [8]. The heteroresistance observed to erythromycin in addition to penicillin can thus be explained by the co-localisation of *pbp2m* and *ermX* on a single genetic element, which may have been amplified as described previously [13].

## Discussion

In 2023, outbreaks of diphtheria were declared in several West African countries. Nigeria has been experiencing a large outbreak, with 13,416 suspected cases since 9 May 2022 [14-16]. Guinea has also declared an outbreak that started in early July 2023, with 538 cases by 13 October [17]. Cases have also been reported in Mali and Niger [18]. A low diphtheria vaccination rate, exacerbated following disruption of public health prevention and response due to COVID-19 pandemic, is an important driver of diphtheria outbreaks. In Nigeria, widespread resistance to benzylpenicillin, ciprofloxacin and trimethoprim-sulfamethoxazole was reported [14], but the levels and mechanisms of resistance remain so far unknown. A possible genetic link between outbreak strains in different West African countries has not been investigated.

Despite nearly one year from the outbreak declarations in West Africa [15], little information is available on the circulating isolates and their genomic or phenotypic characteristics. Travel-related cases from West Africa to metropolitan France in 2023 may provide a window on the ongoing outbreaks. Here, we showed that eight of 10 unlinked isolates were resistant to multiple antimicrobial agents. Gene *pbp2m* typically confers low-level resistance to penicillin and amoxicillin [8]. In our previous global genomic survey, we reported *pbp2m* in less than 5% of non-duplicate *C. diphtheriae* isolates [9]. Here, six of 10 isolates were carrying *pbp2m*, a remarkable observation given that these isolates belong to ten distinct genome-defined types. This surprising result suggests a high prevalence of *pbp2m* in West Africa and might even indicate an African origin of *pbp2m* in *C. diphtheriae*. Gene *ermX* also appeared to be common in West Africa and was always associated with *pbp2m* herein. Mobile elements carrying both *pbp2m* and *ermX* should be the object of enhanced surveillance and further research, especially given their possible genetic amplification upon antimicrobial exposure.

Multidrug resistance is of concern, as it affects negatively clinical care and prophylactic effectiveness to control further disease and spread. Here, resistance was observed both in *tox*-positive and *tox*-negative isolates. The latter isolates were detected by taking samples from cutaneous infections and using mass spectrometry technology to identify bacterial colonies isolated from skin lesions, i.e. not only from classical respiratory diphtheria. Although cutaneous *C. diphtheriae* infections are not typically detected in outbreak settings where diagnosis is mainly based on classical clinical signs of respiratory diphtheria, infected wounds are considered as important for transmission of diphtheria [5].

The data presented here may be of relevance for the ongoing resurgence of diphtheria in West Africa. However, a limitation of this study is the small number of *C. diphtheriae* isolates and uncertainty whether they are representative of the main genotypes circulating in West Africa. It is urgent that more isolates, representative of the ongoing outbreaks, are characterised to guide appropriate antimicrobial therapy and prophylaxis strategies. In addition to representing a potential health threat to West African populations, circulation of multidrug-resistant *C. diphtheriae* is of broader concern and underlines the need to maintain high vaccination coverage even in low-incidence regions, which face the risk of case importation. Despite this, the risk of dissemination within high-vaccination countries seems limited. In France in 2023, no secondary cases were reported, as also observed for cases imported from other areas in 2022 [9].

## Conclusion

Diphtheria, a largely forgotten disease, seems to be re-emerging predominantly in vulnerable populations with low vaccination rates. Furthermore, it presents an additional risk due to the emergence of multidrug-resistant strains. There is an urgent need to reinforce diagnostic laboratory capacity for diphtheria to guide clinical and public health response and to implement microbiological methods including genomic sequencing to decipher transmission patterns within countries, genetic support for resistance, and the links between outbreaks at regional scale.
